# RAC1 Takes the Lead in Solid Tumors

**DOI:** 10.3390/cells8050382

**Published:** 2019-04-26

**Authors:** Pradip De, Jennifer Carlson Aske, Nandini Dey

**Affiliations:** 1 Translational Oncology Laboratory, Avera Cancer Institute, Sioux Falls, SD 57105, USA; Pradip.De@avera.org (P.D.); Jennifer.Aske@avera.org (J.C.A.); 2 Departmental of Internal Medicine, SSOM, University of South Dakota, Sioux Falls, SD 57105, USA

**Keywords:** RAC1, sub-cellular signaling, solid tumors, resistance

## Abstract

Three GTPases, RAC, RHO, and Cdc42, play essential roles in coordinating many cellular functions during embryonic development, both in healthy cells and in disease conditions like cancers. We have presented patterns of distribution of the frequency of RAC1-alteration(s) in cancers as obtained from cBioPortal. With this background data, we have interrogated the various functions of RAC1 in tumors, including proliferation, metastasis-associated phenotypes, and drug-resistance with a special emphasis on solid tumors in adults. We have reviewed the activation and regulation of RAC1 functions on the basis of its sub-cellular localization in tumor cells. Our review focuses on the role of RAC1 in cancers and summarizes the regulatory mechanisms, inhibitory efficacy, and the anticancer potential of RAC1-PAK targeting agents.

## 1. Prologue 

GTPases act as molecular switches. In the GTP (Guanosine triphosphate)-bound active form, RAC1 interacts with effector molecules to initiate signaling, while in the GDP (Guanosine diphosphate)-bound inactive form the signal is terminated. In the unstimulated condition, RAC GTPase exists as a complex with GDP dissociation inhibitor in the cytosol. When activated through the action of soluble chemoattractant, chemokines, phagocytic particles, or any upstream regulator, RAC dissociates from GDI (guanine nucleotide dissociation inhibitors), GDP is exchanged for GTP through the action of membrane-localized guanine nucleotide exchange factors, and RAC, now in its GTP-bound active form, becomes membrane-associated [1,2]. Over 80 GEFs (guanine nucleotide exchange factors) and more than 70 GAPs (GTPase-activating proteins) have been reported, suggesting that Rho-family GTPase regulation is complex and that activity and localization can be modulated by a multitude of signaling pathways depending on the spatiotemporal context [3]. All three GTPases (RHO, RAC, and Cdc42) have been shown to be activated at the front of migrating cells, but the spatiotemporal coordination between them is unknown [4]. RAC1 (Ras-related C3 botulinum toxin substrate1), one of the best characterized RHO GTPases, is an established effector of receptors and an important hub in signaling networks critical for tumorigenesis and metastasis. The RAC1 gene is present on chromosome 7 (7p22) and comprises 7 exons over a length of 29KB [5]. RAC1, but not RAC2 or RAC3 genes, contains an additional exon 3b that is included by alternative splicing in the variant of RAC1b, a constitutively active mutant that is expressed primarily in colon and breast cancer [6]. RAC1 is ubiquitously expressed, and its inactivation by gene targeting in mice leads to embryonic lethality [7].

RAC1 hyper activation is common in human cancers and could be the consequence of overexpression, abnormal upstream inputs, deregulated degradation, and/or anomalous intracellular localization. More recently, cancer-associated gain-of-function mutations in RAC1 have been identified which contribute to tumor phenotypes and confer resistance to targeted therapies [8]. Deregulated expression/activity of RAC guanine nucleotide exchange factors responsible for RAC activation has been largely associated with a metastatic phenotype and drug resistance.

## 2. RAC1 and Solid Tumors

**Figure 1:**
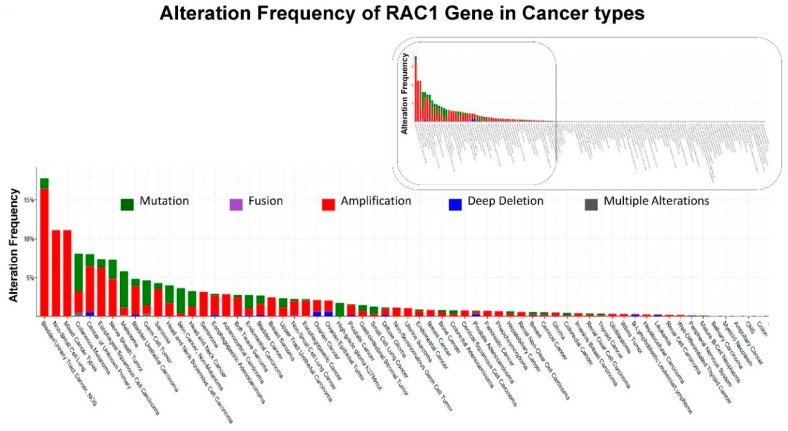
Alterations of RAC1 Gene: Data has been queried from cBioPortal. Figure 1 shows the alteration frequency of RAC1 gene in different cancer types: Data obtained from c-BioPortal (January 2019) representing a combined study of 74247 samples, querying 71857 patients/74247 samples in 240 studies (http://www.cbioportal.org). Y-axis represents alteration frequency of RAC1 gene (colors represent the type of alterations as shown in the figure). The frequency of alterations is sorted on X-axis according to cancer types. Inset shows the entire distribution of the frequency of alterations of the RAC1 gene while the boxed portion of the inset represents only the cancers where the frequency of alterations occurred (boxed in the inset).

Unlike RAS proteins, which are frequently mutated in cancer (around 30%), RAC proteins (RAC1, RAC2 or RAC3) themselves are generally not found to be mutated in cancers at such a high frequency. Both typical Rho GTPases (RhoA, RAC1 and Cdc42 which cycle between an active GTP-bound and inactive GDP-bound conformation and are regulated by GEFs (guanine nucleotide exchange factors), GAPs (GTPase-activating proteins), and GDIs (guanine nucleotide dissociation inhibitors)) and atypical Rho GTPases (GTPase-deficient mutations and those that generally do not hydrolyze GTP) contribute to cancer progression. The “gain-of-function” substitution mutation of RAC1 (e.g., P29S) maintains its intrinsic GTP-hydrolysis property and acquires an inherent GDP-GTP nucleotide exchange capability leading to its “spontaneously activated” state. RhoA or RAC1 are mutated in a few cancers, but in most cancers, expression levels and/or activity of Rho GTPases is altered via a number of regulatory mechanisms [9]. Figure 1 shows the alteration frequency of RAC1 gene in different cancer types. The data was obtained from cBioPortal (January 2019) representing a combined study of 74247 samples. The presence of a long-tail and absence of alteration of RAC1 in more than half of the cancer types is the characteristic feature of alterations of the RAC1 gene in cancers. Bladder/urinary tract cancers, lung cancers, and melanoma are the cancers among which a higher frequency of alterations of the RAC1 gene has been identified, in contrast to other solid tumors like breast, colorectal, and endometrial cancers. 

Recent studies, enabled by next-generation exome sequencing, report activating point mutations in RAC1 GTPases as driver mutations in melanoma, as well as breast, head, and neck cancers. Aberrant activation of RAC1 is implicated in numerous aspects of tumor development and progression and is the subject of several recent peer-reviewed articles [10,11,12,13]. Recently, a recurrent somatic missense mutation at codon 29 of RAC1 in the highly conserved switch I domain that results in the substitution of proline to a serine residue (RAC1 P29S) was discovered in up to 9% of sun-exposed melanomas. This discovery makes RAC1 the third most commonly mutated (somatic) proto-oncogene in melanoma after BRAF and NRAS [14,15,16]. Importantly, the RAC1 P29S mutation was more frequent in melanomas that were wild type for both NRAS and BRAF. There was a similar frequency of the RAC1 P29S mutation in primary (9.2%) and metastatic tumors (8.6%), which is consistent with this mutation occurring early in tumorigenesis. RAC1 P29S was significantly more prevalent in male patients (present in 12.8% of males, compared to 2.4% of females, *P* = 0.01) [14], which is consistent with these mutations being induced by UV exposure. Biochemical and cell-based assays demonstrated that RAC1 P29S is activated and has increased binding activity toward RAC1 effectors, and expression of mutant protein confers increased cell proliferation, altered cell migration, and stimulated membrane ruffling and MAPK signaling [17]. Activating mutations have also been identified in other RAC family members, such as RAC2-P29L and RAC2-P29Q [18]. The COSMIC database show that more than one RAC1 mutation can occur in different cancers types, which includes the large intestine, cervix, liver, endometrium, stomach, esophagus, lung, upper aero-digestive tract, hematopoietic/lymphoid, and breast. The MSK-IMPACT Clinical Sequencing Cohort, which is the most recent large-scale genomic study by the Memorial Sloan-Kettering Cancer Centre that sequenced tumors from more than 10,000 patients, identified many hotspot mutations involving the P29 residue (e.g., P29S, P29F, P29L, and P29T) in melanoma, Merkel cell carcinoma, squamous cell carcinoma, anaplastic thyroid cancer, and breast invasive ductal carcinoma using the cBioPortal [19,20,21]. Although the RAC1 P29S mutation is oncogenic and biochemically active, its clinical relevance in melanoma remains unclear. It has been recently demonstrated that shortening of the 3′ untranslated regions (3′UTR) of mRNA is an important mechanism for oncogene activation including RAC1. Chen et al. recently demonstrated that short 3’UTR isoform of RAC1 substantially upregulated RAC1 expression by escaping from miRNA-targeted repression and played an essential oncogenic role in urothelial carcinoma of the bladder pathogenesis [22].

**Figure 2:**
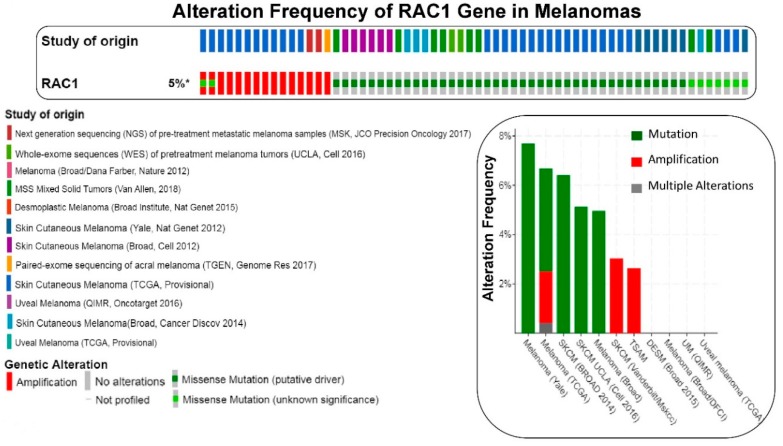
The frequency of alteration of RAC1 gene in melanomas: The oncoprint presents data obtained from c-BioPortal (February 2019) representing a combined study of 1315 samples (http://www.cbioportal.org), querying 1273 patients/1315 samples in 12 studies, as shown in the different color codes of the oncoprint (upper panel) under “Study of origin” in the figure. The percentage (5%*) represents the alteration of the RAC1 gene in patients with melanomas. The lower panel of the oncoprint represents the types of alterations of RAC1 gene in samples as shown under “Genetic Alteration” in the figure. Unaltered cases (RAC1 gene) were not included in the oncoprint. The bar diagram (figure on the right panel) represents the frequency of alterations in RAC1 gene in a few individual melanoma studies where alterations were identified. The Y-axis of the bar diagram represents the alteration frequency of the RAC1 gene (colors represent the types of alterations as shown in the figure). The frequencies of the alterations are sorted on the X-axis according to “Cancer Study”. MSS Mixed Solid Tumors (Van Allen, 2018) is not represented in the bar-diagram.

**Figure 3:**
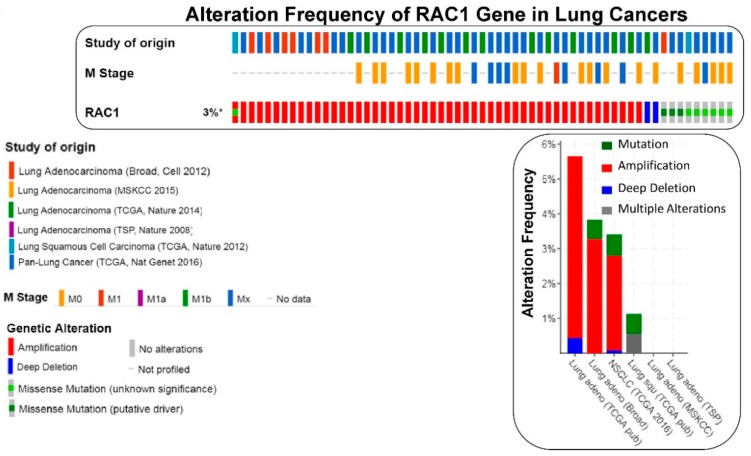
The frequency of alteration of the RAC1 gene in lung cancers: The oncoprint presents data obtained from c-BioPortal (February 2019) representing a combined study of 1933 samples (http://www.cbioportal.org), querying 1933 patients/samples in six studies as shown in the different color codes of the oncoprint (upper panel) under “Study of origin” in the figure. The percentage (3%*) represents the alteration of the RAC1 gene in patients with lung cancers. The lower panel of the oncoprint represents the types of alterations of RAC1 gene in samples as shown under “Genetic Alteration” in the figure. The middle panel of the oncoprint presents distribution of metastatic stage (M Stage as shown in the figure) of the patients where alteration of the RAC1 gene was identified. Unaltered cases (RAC1 gene) were not included in the oncoprint. The bar diagram (figure on the right panel) represents the frequency of alterations in the RAC1 gene in a few individual lung cancer studies where alteration was identified. The Y-axis of the bar diagram represents alteration frequency of the RAC1 gene (colors represent the type of alteration as shown in the figure). The frequencies of alterations are sorted on the X-axis according to “Cancer Study”.

**Figure 4:**
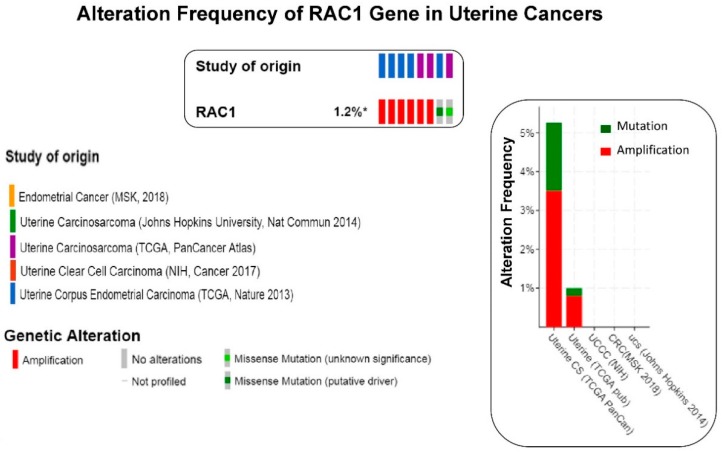
The frequency of alteration of RAC1 gene in uterine cancers: The oncoprint presents data obtained from c-BioPortal (February 2019) representing a combined study of 792 samples (http://www.cbioportal.org), querying 784 patients/792 samples in five studies as shown in different color codes of the oncoprint (upper panel) under “Study of origin” in the figure. The percentage (1.2%*) represents the alteration of the RAC1 gene in patients with uterine cancers. The lower panel of the oncoprint represents the types of alterations of RAC1 gene in samples as shown under “Genetic Alteration” in the figure. Unaltered (RAC1 gene) cases were not included in the oncoprint. The bar diagram (figure on the right panel) represents the frequency of alterations in the RAC1 gene in a few individual uterine cancer studies where alteration was identified. The Y-axis of the bar diagram represents alteration frequency of the RAC1 gene (colors represent the type of alteration as shown in the figure). The frequencies of alterations are sorted on X-axis according to “Cancer Study”.

We have presented alteration frequencies of RAC1 gene in melanomas, lung cancers, and uterine cancers as queried from the cBioPortal (http://www.cbioportal.org). Figure 2 shows the frequency of alteration of the RAC1 gene in melanomas. The oncoprint presents data obtained from cBioPortal (February 2019) representing a combined study of 1315 samples (http://www.cbioportal.org; querying 1273 patients/1315 samples in 12 studies). The bar diagram represents the frequency of alterations in the RAC1 gene in a few individual melanoma studies where alterations was identified. Figure 3 shows the frequency of alteration of the RAC1 gene in lung cancers. The oncoprint presents data obtained from cBioPortal (February 2019) representing a combined study of 1933 samples (http://www.cbioportal.org). The oncoprint represents the types of alterations of the RAC1 gene in samples as shown under “Genetic Alteration” in the figure and the distribution of metastatic stages of the patients where alterations of the RAC1 gene was identified. The bar-diagram represents the frequency of alterations in the RAC1 gene in a few individual lung cancer studies where alterations was identified. Figure 4 shows the frequency of alteration of the RAC1 gene in uterine cancers. The oncoprint presents data obtained from cBioPortal (February 2019) representing a combined study of 792 samples (http://www.cbioportal.org). The oncoprint represents the types of alterations of the RAC1 gene in samples as shown under “Genetic Alteration” in the figure. The bar diagram represents the frequency of alterations in the RAC1 gene in a few individual uterine cancer studies where alteration was identified. It is evident from the data that although the predominant alteration in RAC1 gene is amplification (Figure 1, Figure 3, and Figure 4), melanoma represents cancer wherein most of the alterations observed are mutations of the RAC1 gene (Figure 2). In summary, Figure 1 demonstrates that alteration in the RAC1 gene occurs in only a few of the organ-type cancers, and the frequency never reaches more than 15%. Furthermore, the predominant form of alteration is the amplification (as in bladder and urinary tract cancer) of the gene, followed by mutation (as in melanoma and germ cell tumor). Figure 2 shows the predominant form of alteration occurring in melanoma is mutation (maximum 7.5%). It also shows that the predominant form of the alteration is center-dependent or the study of origin. In contrast to melanoma, amplification of the RAC1 is predominant in lung adenocarcinoma (Figure 3). Interestingly, both amplification and mutation of the RAC1 gene occur in uterine cancers (Figure 4). In melanoma, lung, and uterine cancers, although the percentage of total alteration of the RAC1 gene is around 5–7%, the type of alteration varied depending on the organ-type. 

## 3. Sub-Cellular Location-Based Activation and Regulation of RAC1 Functions in Tumor Cells

Cellular processes orchestrated by RAC1 in tumor cells are achieved via the spatiotemporal activation of RAC1 and the regulation of RAC1 activity, switching between active and inactive states at various subcellular locations, including the plasma membrane, nucleus, and mitochondria [23,24]. This compartmentalization of RAC1 signaling is regulated by a large number of proteins that fine-tune RAC1 functions by promoting/reducing its recruitment, activation, and stability, as well as its deactivation by its regulators via various posttranslational modifications. For example, localization of GEFs and GAPs has been identified as one of the modes of regulation of RAC1 activity. Regulation of plasma membrane-associated processes such as lamellipodia formation and membrane ruffling [25,26], focal contact formation [26], and E-cadherin-mediated cell-to-cell contacts [27] by RAC1 in tumor cells is one of the earliest known functions of RAC1 which occurs through its recruitment and subsequent regulation at the plasma membrane. 

RAC1 function is initiate following its localization to the plasma membrane. Interaction of negatively charged PIP2/PIP3 with the positively charged polybasic sequence of small GTPases targets small GTPases to the plasma membrane [28]. In migrating cells, RAC1 localizes at the leading edge following its interaction with PIP3 [29]. Furthermore, phosphatidylserine is a bona fide binding partner of the polybasic region of RAC1 [30]. RAC1 can be brought to its site of activation, the plasma membrane, upon canonical activation of integrin(s), receptor tyrosine kinases (RTKs), and scaffold proteins [31]. In colon cancer cells, the transmembrane protein LGR5 recruits IQGAP together with RAC1 to the plasma membrane to enhance cell-to-cell adhesion [32]. LGR5 is a target gene of the Wnt-beta-catenin pathway. One of the most frequently mutated oncogenes found in hepatocellular carcinoma and colorectal cancers is beta-catenin. In addition to geranylgeranyl type of prenylation, RAC1 also undergoes reversible palmitoylation at cysteine-178, which eventually stabilizes RAC1 at actin cytoskeleton-linked membrane regions during spreading and migration [33]. Gradients of RAC1 nanoclusters support spatial patterns of RAC1 signaling [34]. Recently, Remorino et al. reported that RAC1 forms nanoclusters of 50–100 molecules which are increased upon RAC1 activation and through interaction with GEFs, GAPs, and effectors via the polybasic region at the plasma membrane of migrating fibroblasts [34]. This selective lipid sorting of small GTPases to form nanometer-scale lipid domains on the plasma membrane has been shown to drive RAC1 signaling [35].

RAC1 localization in the nucleus can be RTK mediated. Activated ERK following EGFR stimulation phosphorylates RAC1 at threonine108 leading to increased nuclear RAC1 [36]. Nuclear localization (nuclear localization signals identified in the C-terminal polybasic region of RAC1) of RAC1 is cell cycle dependent. High RAC1 is observed in the nucleus in late G2 phase, and nuclear exclusion of RAC1 occurs during early G1 phase [37]. RAC1 is also found to localize to centrosomes at G2 phase, prophase, and early prometaphase regulating centrosome separation and mitotic entry [38,39]. Nuclear RAC1 directly interacts with nucleophosmin1 (NPM1), which in addition to acting as a RAC1 chaperone also forms a nuclear protein complex with RAC1, its GEF, ECT2, and the nucleolar transcription factor to promote ribosomal DNA transcription during KRAS-TP53-induced lung tumorigenesis [40]. In the nucleus of colorectal cancer cells, TIAM1, a RAC1-GEF, negatively regulates YAP/TAZ transcriptional activity, hence suppressing their invasiveness [41]. Nuclear accumulation of RAC1-beta-catenin complex following Wnt or RAC1 activation helps in the formation of nuclear beta-catenin-lymphoid enhancer factor (LEF-1) complexes, necessary for transactivation of Wnt-dependent genes [42]. Nucleocytoplasmic shuttling of RAC1 has been reported to drive nuclear shape changes and tumor invasion [43]. Nuclear accumulation of RAC1 increases nuclear plasticity leading to a depletion of cytoplasmic, active RAC1 with a concomitant increase in RhoA signaling driving actomyosin-mediated cell shape changes; the two properties combine to enhance the tumor cell’s invasiveness [44].

RAC1 interacts with BCL2 (a co-localization and physical interaction between the two proteins) at the mitochondrial membrane of BCL2-overexpressing B-cell lymphoma cells [45], which stabilizes the anti-apoptotic, BCL2-mediated superoxide production. Inhibition of the BCL2-RAC1 interaction or blocking superoxide production, sensitizes lymphoma cells to apoptosis [45]. Interestingly, such interaction was only reported in primary cells derived from patients with B-cell lymphoma overexpressing BCL2 but not in noncancerous tissue.

## 4. Cellular Signaling of RAC1 in Solid Tumors

**Figure 5:**
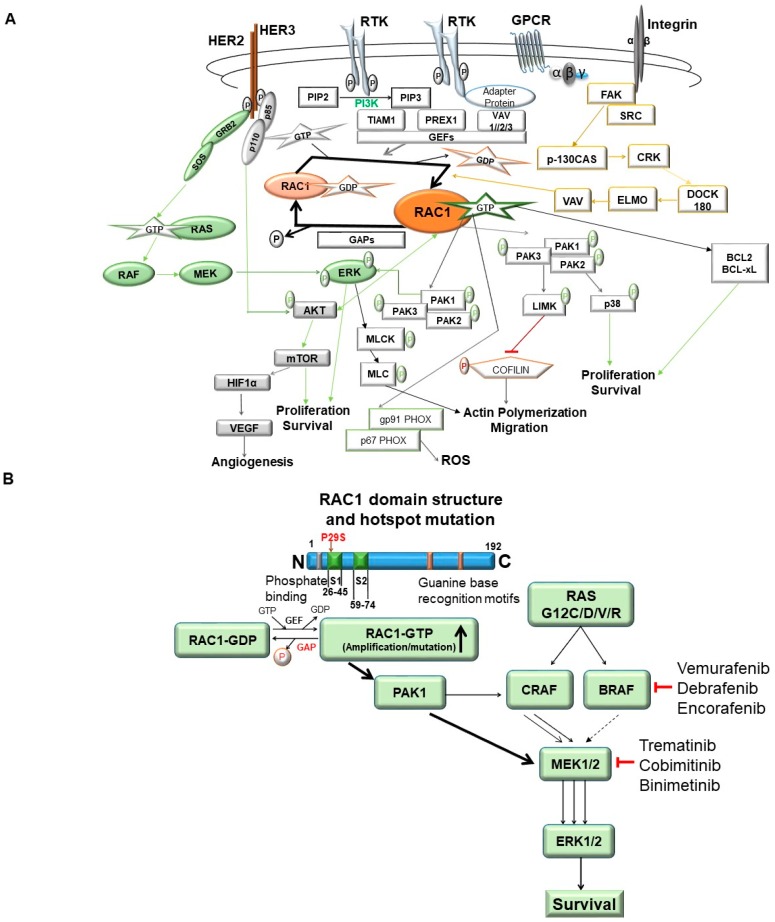
RAC1 signaling in tumor cells: RAC1 signaling in tumors cells and the involvement of the RAC1 protein in tumorigenesis and drug-resistance as presented in the review. (**A**) represents the RAC1 signaling pathway. RAC1 cycles between an inactive-GDP-bound form, and an active-GTP-bound form. Signals from cell surface receptors, for example, G-protein coupled receptors (GPCRs), receptor tyrosine kinases (RTKs), and integrins (alpha, beta) converge on the guanine nucleotide exchange factors (GEFS: VAV, TIAM, PREX), which converts RAC1 into its active state. Once activated, RAC1 can bind to a wide range of effectors, which in turn influence both a variety of oncogenic phenotypes like proliferation/survival, actin remodeling/migration, metastasis, and angiogenesis in tumor cells. In the process of functions mentioned above within tumor cells, RAC1 signals are closely knit to both the PI3K-AKT and the RAS-MAPK pathways integrating extracellular stimulations with oncogenic alterations. Other mechanisms, such as the stabilization of the active form of RAC1 by sumoylation or sequestration of the inactive form by guanine nucleotide dissociation factors (GDIs), can also influence the level of RAC1 signaling. Schematic illustration of RAC1 signaling pathways and functions of its effectors are presented. Green font represents signaling events following phosphorylation-mediated activation of the protein. The red font represents signaling events following phosphorylation-mediated inactivation of the protein. The blue border represents adaptor protein, the orange border represents GDP state/inactivating phosphorylation, the green border represents activated phosphorylation, the yellow border represents metastatic pathway, the yellow arrows represent metastatic pathway network, the green arrows represent proliferation/survival network, and the red line represents inhibition of signals. (**B**) illustrates the involvement of RAC1 in tumorigenesis and drug-resistance: RAS signaling-mediated tumorigenesis and the RAC1-PAK1 pathway-mediated resistance against RAS–RAF-MEK pathway-targeted drugs in cancers has been illustrated. RAC1 plays a cardinal role in the development of drug-resistance, especially in BRAF-mediated cancers like melanoma in which the development of resistance to BRAF inhibitors like vemurafenib, debrafenib, and encorafenib for a certain time leads to the hyper-activation of RAC1. The hyper-activation of RAC1 may either occur in the presence of upstream RTK-activation or due to the accompanying amplification or hot-stop mutation of RAC1. Under hyper-activation of RAC1, the RAC1-GTP activates PAK1, leading to the downstream activation of MEK and bypassing the upstream BRAF inhibition.

RAC1 GTPases, small G-proteins widely implicated in tumorigenesis and metastasis, transduce signals from receptor tyrosine-kinases (e.g., EGFR/ HER2) or non-receptor tyrosine kinases (e.g., SRC, FAK), G-protein-coupled receptors (GPCRs), and integrins (e.g., alpha/beta) and control a number of essential cellular functions, including motility, adhesion, and proliferation. Figure 5A illustrates RAC1 signaling pathways and functions of its effectors. Deregulation of RAC1 signaling in cancer is generally a consequence of enhanced upstream inputs from tyrosine-kinase receptors, PI3K, or GEFs (e.g., VAV, PREX, TIAM) or reduced RAC1 inactivation by GAPs (e.g., RhoGAP). In breast cancer cells RAC1 is a downstream effector of ERBB receptors via GRB7-VAV2 and mediates migratory responses by ERBB1/EGFR ligands, such as EGF or TGFα, and ERBB3 ligands, such as heregulins [46]. Recent developments in the field led to the identification of the RAC1-GEF PREX1 as an essential mediator of RAC1 responses in breast cancer cells. PREX1 is activated by the PI3K product PIP3 and Gβγ subunits and integrates signals from ERBB2 receptors and GPCRs. Most notably, PREX1 is highly overexpressed and amplified in human luminal breast tumors, particularly those expressing ERBB2 and estrogen receptor (ER). The HER2-PREX1-RAC1 signaling pathway may represent an attractive target for breast cancer therapy [47]. Similarly, RAC1 GTPase was found to signal the Wnt-beta-catenin pathway-mediated tumor cell phenotypes in triple negative breast cancers [48].

The most established mechanism for RAC1-mediated cytoskeleton reorganization is through PAK (p21-activated kinase), a family that comprises six isoforms (PAK1–6). PAKs have an N-terminal GTPase binding domain (GBD) where RAC1-GTP or Cdc42-GTP binds and a C-terminal serine/threonine kinase domain. Binding of active Cdc42 or RAC1 to the GBD domain of group I PAKs (PAK 1, 2, and 3) releases auto-inhibition and enhances kinase activity. Overexpression and/or hyperactivation of PAK isoforms have been detected in several cancer types, including breast cancer [49]. PAK1 is implicated in the regulation of cytoskeleton dynamics and cell motility, transcription, survival, and cell-cycle progression and also activates RAF-MEK signaling [50]. Upregulation of RAC1-PAK1 signaling exerts its effects on cytoskeleton organization, ROS production, and proliferation/survival through phosphorylation of various proteins, including LIMK (LIM kinase)/co-filin, gp91PHOX/p67 PHOX and AKT/BCL2/BCL-xL respectively [51,52,53] (see Figure 5A).

## 5. RAC1 Signaling in Tumor Cell Proliferation

Several studies, including ours [48], demonstrated that RAC1 GTPase is upregulated/hyperactivated in breast cancer cells (in all three subsets like luminal, HER2-enriched, and TNBC) and is associated with poor prognosis. Studies by Hein et al. revealed that RAC1-GTP activity is necessary for G2/M cell cycle activation and cell survival in response to ionizing radiation of breast cancer cells. Using a RAC1-specific inhibitor (NSC23766, albeit in high concentration, 100 µM) and the dominant negative mutant N17RAC1, they demonstrated that RAC1 inhibition decreased the phosphorylation of ERK1/2 and IκBα, as well as the protein expression levels of BCL-xL and MCL-1 protein in the HER-selected breast cancer cells along with clonogenic growth survival [54]. Active RAC1 was also required for RICTOR-mTORC2-dependent invasion and motility [55], essential for metastasis. Phosphatidylinositol 3,4,5-triphosphate (PIP3) also plays an important role in the activation of guanine nucleotide exchange factors for the activation of small GTPases, including RAC1. GEFs (e.g., VAV1/2/3, PREX1, TIAM) contain a PH domain and once they are bound to PIP3 (in the membrane) enhance small GTPase activity [56]. Although VAV1 alterations have been historically regarded as predominantly associated with hematopoietic malignancies, certain oncogenic mutation(s) of VAV1 in solid tumors have also been reported [57,58]. VAV1 mutations have been identified in human cancers of various origins in the context of different oncogenic phenotypes. Recently, Shalom et al. tested the transforming potential of three mutations (E59K, D517E, and L801P) reported in human lung adenocarcinoma. Studies demonstrated that mutations like E59K and D517E are highly transforming indicating that specific mutations of VAV1 can be recognized as a bona fide oncogene in human cancers [59]. GAPs are multi-domain proteins characterized by the presence of various functional domains including the conserved GAP domain which is triggering the intrinsic GTP hydrolysis. Increased GDP→GTP nucleotide exchange by RAC1-GEF in the context of the event of the activation of RAC1 mediates oncogenic effects, while alterations in RAC1-GAPs are not generally cancer associated [47,60]. Overexpression and/or hyperactivation of RAC1’s immediate effector molecule, PAK, has been detected in several cancer types, including in breast cancer [49]. PAK1 is implicated in the regulation of cytoskeleton dynamics and cell motility, transcription, survival, cell-cycle progression, and resistance to hormonal therapy (e.g., tamoxifen) in ER+ breast cancer [48,61]. NF2-knockout mouse embryonic fibroblasts (MEF) exhibit increased RAC1 activity (RAC1 activity is inversely regulated by NF2), loss of contact inhibition, and significantly increased canonical Wnt signaling [62]. 

RAC1 controls cancer cell invasion by regulating the production of matrix metalloproteinases, MMPs (RAC1/PAK1/p38/MMP-2 axis regulated angiogenesis), and their natural inhibitors, the tissue-specific inhibitors of MMP (TIMPs) [63,64]. Degradation of the extracellular matrix (ECM) by MMPs is essential for this kind of collective cell migration [65,66]. It is also known that colorectal cancer cells express an alternatively spliced RAC1b and depend on RAC1b signaling for survival via NF-kB activation [67]. Other studies found that RAC1b increased cell-cycle progression of G1-S phase and survival and transformation of NIH3T3 fibroblasts [68,69], promoted epithelial-mesenchymal transition of mouse mammary epithelial cells [70], and supported the further stimulation of Wnt signaling in HCT116 colorectal cells [71]. In a conditional lung cancer mouse model, RAC1 function was needed for KRAS-mediated cell proliferation and tumorigenicity [72]. Similarly, mice lacking the RAC-specific GEF Tiam1 are protected from RAS-driven skin cancer, developing fewer tumors [73]. These results strongly indicate that RAC1-GTP has the capacity to regulate tumorigenicity and increase tumor cell proliferation even in RAS-driven cancers.

## 6. RAC1 Signaling in Tumor Cells Migration

The small GTPase RAC1 has been implicated in the rearrangement of the actin cytoskeleton and remodeling of the plasma membrane in response to extracellular stimuli, a process known as “membrane ruffling” [25]. The RAC/RHO GTPases have been found to play a role in signaling that activates a variety of transcription factors, cell cycle progression, and integrin-mediated cell migration [74,75,76]. Because aberrant RAC1 GTPase signaling activities are widely associated with human cancer, key components of RAC1 GTPase signaling pathways have attracted increasing interest as potential therapeutic targets.

Hall, Ridley, and Nobes demonstrated that the RAC1 initiates lamellipodia formation in downstream of PDGF stimulation in contrast to the fact that RhoA stimulates the formation of contractile actomyosin fibers (i.e., stress fibers) following LPA signaling [25], and Cdc42 promotes filopodia [26]. RAC1-mediated lamellopodia formation involves binding to the SCAR/WAVE regulatory complex (WRC) components of Sra1 and WAVE1. An effector molecule ELMO, a downstream of RhoG, controls RAC-driven actin remodeling and migration [77,78,79]. Epithelial–mesenchymal transition (EMT) is one of the cardinal features associated with the metastatic process in tumor cells wherein these cells lose their polarity, shape (especially in the case of epithelial tumors; from epithelioid to mesenchymal), and cell-to-cell adhesion in order to acquire migratory and invasive potential as well as stem-like features [80]. Understandably, RAC1 plays an important role in EMT by virtue of its property to regulate cell polarity, migration, invasion, and stemness [81] in different cancers including gastric adenocarcinoma [82], squamous lung cancer [83,84], and colorectal cancers [85]. A mechanism involving RAC1 has been reported to involve the PI3K/AKT-RAC1-JNK axis in gastric adenocarcinoma [82], PI3K in squamous lung cancer [83,84], and the activation of STAT3 in colorectal cancer [85], highlighting the direct association of RAC1 activation and tumor aggressiveness.

## 7. RAC1 Signaling in Tumor Angiogenesis and Resistance

Tumor-induced angiogenesis supplies necessary nutrients and fosters tumor growth [86]. RAC1 is involved in angiogenesis and required for vascular integrity and the sprouting blood vessels (associated with tumor-induced angiogenesis), as demonstrated in a conditional RAC1 knockout mouse model [87]. RAC1 is activated by various angiogenic factors, e.g., vascular endothelial growth factor (VEGF)-A, angiopoietin 1, basic fibroblast growth factor (FGF), and others [88]. Activation of RAC1 and also RAC2 in endothelial cells regulates adhesion, filopodia formations, morphogenesis, cell proliferation, and integrin-directed endothelial migration [89,90,91,92]. Van Allen et al., in their clinical study on patients with BRAF V600-mutant metastatic melanoma who received vemurafenib or dabrafenib monotherapy, found early disease progression in 14 patients (out of 45 patients). No patients who demonstrated a sustained response to therapy (≥12 weeks of therapy) exhibited RAC1 P29S mutation [93]. Following these clinical studies, several other investigators have confirmed that the RAC1 P29S hotspot mutation in melanoma may be an important predictor for vemurafenib and dabrafenib resistance in patients [8]. Genetic deletion or pharmacological inhibition of RAC1 in NRAS Q61K-induced melanoma has been shown to suppresses tumor growth, lymph node spread, and tumor cell invasiveness, suggesting a potential value for RAC1 inhibition in this type of tumors [94].

RAC1 has been identified as a major mediator of chemo-resistance [95,96,97]. This role of RAC1 has been suggested in both treatment resistance and compensatory mechanisms following conventional chemotherapy (in diseases including chronic lymphocytic leukemia and squamous cell carcinoma) as well as targeted therapy (anti-EGFR for lung, anti-HER2/estrogen targeted therapies in breast cancers, BRAF protein inhibitors in melanoma, and anti-angiogenic therapies in prostate cancers). Translational laboratory-based data showed that in many cases sensitivity to the targeted therapeutic is restored upon RAC1 inhibition. Figure 5B summarizes the involvement of RAC1 in tumorigenesis and drug-resistance. RAS signaling-mediated tumorigenesis and RAC1-PAK1 pathway-mediated resistance against RAS–RAF pathway-targeted drugs in cancers have been of particular interest. Mechanistic involvement of RAC1 in the development of chemo-resistance, radio-resistance, resistance to targeted therapies and immune evasion opened the opportunities for interfering RAC1 signaling pathway in cancer therapeutics [95].

Furthermore, RAC1 signaling provides a novel insight into the mechanisms of cancer cell survival under ER stress (endoplasmic reticulum stress). N92I mutation of RAC1 is oncogenic in the sarcoma model [18]. RAC1 promotes cell survival under ER stress in cells with an oncogenic N92I RAC1 mutation. Bright et al. have identified a novel connection between the UPR (unfolded protein response) and N92I RAC1, whereby RAC1 attenuates phosphorylation of EIF2S1 under ER stress and drives overexpression of ATF4 in basal conditions. The above-mentioned finding is important since oncogenesis and uncontrolled cancer cell division often induce ER stress, and cell fate under ER stress is controlled by UPR. Their study demonstrated that oncogenic mutant RAC1 and NRAS drive resistance to ER stress by activating MEK/ERK signaling [98]. 

Interestingly, a study from Prof. Lewis Cantley’s laboratory reported that RAC1 drives macropinocytosis of an extracellular protein and regulates the adaptive metabolic pathway. In a subset of non-small cell lung cancer (NSCLC) cell lines (including H1299, H441, H1975, H1781, and HCC4006), it was demonstrated that cells survived in the absence of glucose by internalizing and metabolizing extracellular protein via macropinocytosis. There are glucose-independent cells (glucose-independent NSCLC cells require extracellular protein for growth during glucose withdrawal) wherein macropinocytosis is increased and is regulated by phosphoinositide 3-kinase (PI3K) activation of RAC-PAK signaling [99]. The exact role of RAC1-mediated micropinocytosis in tumorigenesis and drug resistance has not been established.

## 8. Epilogue 

One can expect a merge between the mechanistic evidence for the involvement of RAC1 in the regulation of different tumorigenic phenotypes and the utilization of the evidence-based knowledge in clinics. Studies have provided evidence that RAC1 is involved in tumorigenesis, proliferation, metastatic events, and the development of resistance. RAC1 has been associated with the expression of PD-L1 in melanomas carrying RAC1 P29 mutations. Through reverse phase protein array (RPPA) analysis, Aplin et al. found that PD-L1 was significantly upregulated along with other cell cycle proteins like CYCLIN B1 following RAC1 P29S expression. Western blot and flow cytometry analyses revealed a robust increase in PD-L1 expression, specifically with RAC1 P29S expression. Using the Skin Cutaneous Melanoma (SKCM) database in the TCGA, they also demonstrated that PD-L1 expression was significantly increased in conditions of RAC1 P29S compared to RAC1 WT in melanoma patients [100,101]. Considering the relationship between the functional PD1-PD-L1 axis and the efficacy of immunotherapy drugs, PD-L1 positive tumors (melanomas) expressing oncogenic RAC1 P29S hotspot mutation may be a suitable situation to test immunotherapy drugs. A number of inhibitors of RAC1 are being tested in several laboratories. PAK1 is identified as an interactor of the Rho GTPases RAC1 and Cdc42. PAK1 was later shown to play a diverse role in cell signaling using its catalytic and scaffolding activities [102]. The consensus from published studies is that targeting PAK proteins provides the best potential treatment for RAC1 mutant melanomas. The first structure-based virtual screening led to the discovery of NSC23766, the first selective RAC1 inhibitor with high micromolar IC50 [103]. Araiza-Oliviera et al. showed that use of PAK inhibitors (e.g., Frax-1036 and PF3758309) or the MEK inhibitor PD325901 almost completely prevented aberrant embryonic abnormalities in RAC1P29S-injected zebrafish embryos; in contrast, it was only partly prevented with the RAC inhibitor NSC23766 [101]. To date, several PAK1-targeting compounds have been developed as preclinical agents, including one (PF-03758309) that has been evaluated in a clinical trial in a variety of solid tumors (NCT00932126). In the future, it is expected that the scientific knowledge will be put into action in clinics towards designing personalized clinical trials for the therapeutic management of cancers in which a specific involvement of RAC1 has been identified. As GTPase, RAC1 is not an easy target for treatment; studies have been conducted to inhibit its downstream PAK1. Since mutant allele-specific covalent inhibitor of oncogenic variants of KRAS-G12C (not KRAS G12D or KRAS G12V), ARS1620, and MRTX849 are in the clinical development (recently presented at the NCI/NIH-sponsored session titled New NCI Collaborative Initiatives on Rare Tumors and RASopathies, presentation by Prof. McCormick, AACR Annual Meeting, Atlanta 2019), a similar allele-specific covalent inhibitor(s) of oncogenic variants of RAC1-P29S may have a significant clinical application. The chemistry and technical hurdles of the problem are beyond the scope of the review.
